# Nanodiamonds facilitate killing of intracellular uropathogenic *E*. *coli* in an *in vitro* model of urinary tract infection pathogenesis

**DOI:** 10.1371/journal.pone.0191020

**Published:** 2018-01-11

**Authors:** Janaki Kannan Iyer, Alexia Dickey, Parvaneh Rouhani, Anil Kaul, Nirmal Govindaraju, Raj Narain Singh, Rashmi Kaul

**Affiliations:** 1 Department of Biochemistry and Microbiology, Oklahoma State University-Center for Health Sciences, Tulsa, Oklahoma, United States of America; 2 School of Materials Science and Engineering, Oklahoma State University-Tulsa, Tulsa, Oklahoma, United States of America; 3 Health Care Administration, Oklahoma State University-Center for Health Sciences, Tulsa, Oklahoma, United States of America; RMIT University, School of Science, AUSTRALIA

## Abstract

About 25–44% of women will experience at least one episode of recurrent UTI and the causative agent in over 70% of UTI cases is uropathogenic *Escherichia coli* (UPEC). UPEC cause recurrent UTI by evading the bladder’s innate immune system through internalization into the bladder epithelium where antibiotics cannot reach or be effective. Thus, it is important to develop novel therapeutics to eliminate these intracellular pathogens. Nanodiamonds (NDs) are biocompatible nanomaterials that serve as promising candidates for targeted therapeutic applications. The objective of the current study was to investigate if 6 or 25 nm NDs can kill extracellular and intracellular UPEC in infected bladder cells. We utilized the human bladder epithelial cell line, T24, and an invasive strain of UPEC that causes recurrent UTI. We found that acid-purified 6 nm NDs displayed greater antibacterial properties towards UPEC than 25 nm NDs (11.5% vs 94.2% CFU/mL at 100 μg/mL of 6 and 25 nm, respectively; *P<0*.*001*). Furthermore, 6 nm NDs were better than 25 nm NDs in reducing the number of UPEC internalized in T24 bladder cells (46.1% vs 81.1% CFU/mL at 100 μg/mL of 6 and 25 nm, respectively; *P<0*.*01*). Our studies demonstrate that 6 nm NDs interacted with T24 bladder cells in a dose-dependent manner and were internalized in 2 hours through an actin-dependent mechanism. Finally, internalization of NDs was required for reducing the number of intracellular UPEC in T24 bladder cells. These findings suggest that 6 nm NDs are promising candidates to treat recurrent UTIs.

## Introduction

Urinary tract infections (UTIs) account for more than 12.7 million physician and emergency room visits resulting in ~3.5 billion dollars in treatment costs in the US [1, 2]. Women show a higher susceptibility to contract UTIs than men [3, 4]. Currently, there is no effective vaccine available to prevent UTIs and antibiotics remain to be the main line of treatment. Despite antibiotic treatment, it is estimated that anywhere from 25–44% of women will develop at least one episode of recurrent UTI [5]. In more than 70% cases of recurrent UTI, the causative agent is *E*. *coli* [6, 7]. Some strains of uropathogenic *E*. *coli* (UPEC) cause persistent infections by invading bladder epithelium or cells to form quiescent intracellular reservoirs (QIRs) or intracellular bacterial communities (IBCs). These QIRs and IBCs evade immune system surveillance and are not targeted by antibiotics, thereby resulting in recurrent cystitis and pyelonephritis [8, 9]. Also, many of these intracellular UPECs have become resistant to commonly prescribed antibiotics that make the treatment of UTI difficult, thereby increasing the incidence of recurrent UTI [10, 11]. Antimicrobial resistance in UTI-causing micro-organisms is a globally growing threat [12]. Therefore, there is an urgent need to develop novel therapeutics that are directly targeted to reach inside the host cells to kill virulent intracellular bacteria.

Carbon-based nanoparticles, that include nanodiamonds (NDs), are promising candidates for delivering drugs due to their small size, chemically inert core, tunable surface functionality, and their ability to be internalized by mammalian cells [13–16]. NDs are also more biocompatible and show less cytotoxicity in biological systems compared to other carbon-based nanoparticles [17, 18]. The nanoscale size of NDs also provides a large surface area to adsorb or covalently link therapeutic molecules like drugs. There are many reports that have utilized NDs loaded with drugs belonging to the anthracycline family for the treatment of cancers like leukemia, lung, prostate and hepatic cancer [19–22]. NDs can also be tested as candidates for the treatment of infectious diseases. There have been a few reports on the antibacterial effects of plain NDs on Gram-positive and Gram-negative bacteria [23–25]. However, none of the studies have investigated the ability of NDs to kill pathogenic intracellular bacteria like UPEC that can reside in the host cells. Since NDs are internalized in mammalian cells [16, 26], we hypothesized that NDs will be taken up by human bladder cells and facilitate the killing of intracellular pathogens.

In the current study, we tested our hypothesis by evaluating the ability of NDs of two different sizes to be internalized by human bladder cells to target and kill intracellular UPEC. We utilized an *in vitro* UTI model comprising of the T24 human bladder epithelial carcinoma cell line and infected these cells with clinically isolated invasive strain of UPEC that expresses the Dr adhesin. Dr adhesion bearing *E*. *coli* utilize host receptors to attach and internalize into bladder and kidney cells and tissues [27]. Clinical and experimental studies have shown that Dr bearing *E*. *coli* cause cystitis and acute and chronic pyelonephritis [28, 29]. NDs with the average particle size of 6 nm and 25 nm synthesized by the detonation method or the high pressure/high temperature (HPHT) method were utilized in the current study [30]. The two methods of ND synthesis result in NDs with different morphologies, phase purities, defect structures and surface functional groups that can influence their interactions with human cells or bacteria [31].

Commercially obtained 6 and 25 nm NDs were purified by acid treatment to remove metals and other carbon-based contaminants [32]. The phase purity, surface functional groups, surface charge and particle size, morphology and crystallinity of the NDs were characterized by Raman spectroscopy, Fourier transform infrared (FTIR) spectroscopy, zeta potential measurements and transmission electron microscopy (TEM), respectively. We found that 6 nm acid-treated NDs showed better antibacterial activity than 25 nm acid-treated NDs and facilitated the killing intracellular UPEC at concentrations that were non-toxic to T24 bladder cells as compared to acid-treated 25 nm NDs. We further determined that internalization of 6 nm NDs is critical for the reduction of intracellular UPEC in infected bladder cells. The findings from this study will enable us to evaluate the feasibility of using NDs as therapeutic agents to treat UTIs caused by invasive UPEC.

## Materials and methods

### Purification and characterization of NDs

A slurry of NDs with an average size of 25 nm was obtained from Advanced Abrasive Corporation (Pennsauken, NJ) while NDs with an average size of 6 nm was obtained in the powder form from Nanostructured and Amorphous Materials Inc (Houston, TX). The 25 nm ND slurry was heated at 70°C to obtain a powder. The nanodiamonds were purified by acid-treatment as described previously [32]. Briefly, 1 mg of the 25 or 6 nm ND powders were treated with a mixture of H_2_SO_4_ (95%) and HNO_3_ (68%) at a volume ratio of 9:1 at 70°C for 24 hours to remove the carbon impruties. Upon cooling, the acids were decanted and 0.1 M NaOH was added slowly to the NDs until the suspension became basic. The suspension was heated at 70°C for 2 hours to neutralize the surface from sulfuric and nitride ions. Next the supernatant was decanted and 0.1 M HCl was added to the NDs and heated at 70°C for 2 hours to remove metal impurities. Finally, the supernatant was decantated and the NDs were washed with endotoxin free deionized water followed by centrifugation at 14,385 × g for 30 minutes (Allegra X-30 Centrifuge, BECKMAN COULTER). Washing was repeated till the pH of the suppernatant reached 7 and ions were removed. The NDs were air dried at room temperature and characterized.

### Characterization of NDs

#### Raman spectroscopy

Raman spectroscopy was used to confirm the removal of the impurity and presence of the diamond. The sample preparation approach was to mix the ND powder with water, then add few drops of the mixture on a glass slide and let it be dried at room temperature. The dried powder made a flat surface which is desirable for the Raman measurment. Raman spectra were obtained by using a Nicolet Almega XR Dispersive Raman 960 spectrometer (Thermo Fisher Scientific, Waltham, MA) with 532 nm laser excitation over the range 750–2500 cm^−1^ at 80% of incident laser power.

#### Fourier transform infrared spectroscopy (FTIR)

FTIR spectroscopy was used to confirm the functional groups on NDs surface. For FTIR measurement, 1 mg of a ND sample was added to 200 μl of deionized water. The suspension was added to an aluminum coated silicon substrate, and dried at room temperature. FTIR spectra were collected in the reflection mode with an incident beam angle of 70°. FTIR spectra were recorded by Varian 680-IR Fourier Transform Infrared (FT-IR) spectrometer (Agilent Technology) with PIKE VeeMAX II reflection setup.

#### Zeta potential measurement

Zeta potential measurement was used to confirm the surface charges of the NDs. For sample preparation, 1 mg of the ND was mixed with 1 ml of distilled water, then inserted in folded capillary cells. This measurement was performed using Malvern Zeta Sizer ZS90 with 633 nm laser excitation.

#### Transmission electron microscopy (TEM)

TEM imaging was used to study the morphology of the NDs. TEM analysis was performed by air drying NDs on holey carbon film grids. The samples were imaged on a JEOL JEM-2100 transmission electron microscope (JEOL USA, Inc., Peabody, MA).

### Bacterial strain and human cell line maintenance

*Escherichia coli* strain IH1128 (O75:K5: H^-^ strain) is a Dr adhesin bearing clinically isolated uropathogenic strain. Pure cultures were grown on Luria agar plates and routinely tested for hemagglutination as described previously [33]. The human bladder epithelial carcinoma cell line, T24 [34], was generously provided by Dr. Dale E. Bjorling from University of Wisconsin-Madison. T24 bladder cells were cultured and grown in nutrient media comprising of DMEM:F12 (1:1; Thermo Fisher Scientific, Waltham, MA) supplemented with 9% fetal bovine serum (Atlanta Biologicals, Inc., Flowery Branch, GA) and antibiotics (100 U/mL penicillin and 100 μg/mL streptomycin; Thermo Fisher Scientific, Waltham, MA) at 37°C and a humidified atmosphere containing 5% CO_2_.

### Antibacterial assay

*E*. *coli* was grown on Luria agar plates at 37°C. A bacterial suspension was prepared by resuspending bacterial colonies in DMEM/F12 media till the optical density at 600 nm was 0.5 that corresponded to approximately 5 × 10^8^ CFU/mL. The bacterial suspension was diluted 10^4^ times and treated with different concentrations of acid-treated 6 or 25 nm NDs or amoxicillin for 2 hours at 37°C. After treatment, the bacterial suspension was diluted and plated on sterile Luria agar plates following incubation at 37°C for 18 hours. The CFU were enumerated and the CFU/mL of untreated samples (0 μg/mL) was designated as 100%. The CFU/mL of the treated samples were calculated relative to the untreated sample and the % CFU/mL was depicted using GraphPad Prism v7 (GraphPad Software, Inc., La Jolla CA).

### Cell viability assay

Cell viability was evaluated by measuring the reduction of 3-(4,5-dimethylthiazol-2-yl)-2,5-diphenyltetrazolium bromide (MTT) to formazan [35]. The MTT reagent was purchased from Life Technologies (Thermo Fisher Scientific, Waltham, MA). T24 bladder cells were plated on 96-well tissue-culture coated plates for 18 hours followed by treatment with different concentrations (0, 10, 50, 200 or 500 μg/mL) of 25 or 6 nm commercial or acid-treated NDs for 24 hours at 37°C. 5 mg/mL of MTT solution was added 2 hours prior to the completion of the incubation time. After incubation, the cells were washed with 1X phosphate buffered saline (PBS) and lysed with DMSO to solubilize the formazan accumulated in cells. The absorbance of lysates was measured at 540 nm and the absorbance of untreated cells (0 μg/mL) was designated as 100% survival. The absorbance of the treated samples was calculated relative to the untreated sample and the % cell survival was depicted using GraphPad Prism v7 (GraphPad Software, Inc., La Jolla CA).

### Transmission electron microscopy imaging of T24 bladder cells treated with NDs

T24 bladder cells were plated on tissue culture coated 12-well plates for 18 hours followed by treatment with 100 μg/mL of acid treated 6 nm or 25 nm NDs for 4 hours. The cells were washed with 1X PBS, scraped off the tissue culture plate and fixed in 2% glutaraldehyde made in 0.1 M sodium cacodylate buffer pH 7.0. Cells were washed with 60 mM sodium cacodylate containing 180 mM sucrose and treated with 1% OsO_4_ for 1 hour at room temperature. After washing, cells were dehydrated using a series of graded ethanol and finally with propylene oxide. Cells were then embedded in Embed 812 resin and sectioned into 70 nm thick sections using a Reichert-Jung Ultracut E ultramicrotome. The sections were placed on carbon film TEM grids and stained with uranyl acetate and Reynold’s lead citrate. The sections were imaged on a JEOL JEM-2100 transmission electron microscope (JEOL USA, Inc., Peabody, MA).

### Flow cytometry

Acid treated 6 nm NDs were labeled with FITC (Thermo Fisher Scientific, Waltham, MA) based on the manufacturer’s recommendations and similar to a protocol as described previously [36]. Briefly, NDs suspended in borate buffer were incubated with FITC solution prepared in DMSO at room temperature for 1 hour. Following incubation, the suspension was centrifuged at room temperature for 10 minutes at 20,000 g until there was no detectable free FITC. T24 bladder cells were plated on 24-well plates for 18 hours and treated with 0, 50, 100 or 200 μg/mL of FITC-labeled or unlabeled 6 nm acid treated NDs for 1 hour at 37°C. Following treatment with NDs, the cells were washed with 1X PBS at room temperature and dislodged from the wells using a non-enzymatic cell dissociation buffer (Thermo Fisher Scientific, Waltham, MA). The cells were evaluated using an Accuri C6 flow cytometer. The T24 cells were analyzed for changes in FITC fluorescence by FlowJo (FlowJo LLC, Ashland, Oregon).

### Confocal microscopy

T24 bladder cells were plated on coverslips on 12-well tissue culture treated plates for 18 hours followed by treatment with 0, 50, 100 or 200 μg/mL of FITC-labeled 6 nm acid treated NDs for 1 hour at 37°C. The cells were washed with 1X PBS at room temperature, fixed with 4% paraformaldehyde made in PBS and permeabilized with 0.1% Triton X-100. After washing with 1X PBS, the T24 bladder cells were stained with phalloidin labeled with alexa fluor 660 to detect actin and DAPI to detect the nucleus. The coverslips with stained cells were mounted onto slides and observed under a Leica TCS SPE confocal microscope using a 40X objective. For kinetic studies, the cells were treated with 100 μg/mL of NDs for 30, 60 or 120 minutes and processed similarly as described above. For internalization studies involving cytochalasin D, T24 bladder cells were pretreated with 10 μM cytochalasin D for 1 hour following treatment with NDs for 2 hours. The cells were then processed as described above and observed by confocal microscopy. Z-stack analysis was performed in all cases where internalization of NDs was investigated. All images were processed using Adobe Photoshop.

### Bacterial invasion assays to determine number of intracellular bacteria

A modified gentamicin protection assay was performed as described [28]. T24 bladder cells were plated on tissue-culture treated 24-well plates for 18 hours. Cells were infected with Dr fimbriae bearing *E*. *coli* at an MOI of 25:1. The plates were centrifuged at 500 × g using a Beckman Allegra 6R centrifuge. The cells were allowed to incubate with bacteria for 1 hour at 37°C. Gentamicin was added at a concentration of 100 μg/mL for 1 hour at 37°C to kill extracellular bacteria. The infected T24 bladder cells were washed and treated with 0, 50, 100 or 200 μg/mL of 6 or 25 nm acid treated NDs in the presence of 10 μg/mL gentamicin for 24 hours at 37°C. The cells were lysed with 1% Triton X-100 in PBS and cell extracts were plated onto sterile Luria agar plates followed by incubation at 37°C for 18 hours. The bacterial CFUs were counted on the following day that represented *E*. *coli* internalized into T24 bladder cells. The CFU/mL obtained in infected T24 bladder cells treated with 0 μg/mL of NDs was designated 100% of internalized bacteria and the CFU/mL of all other samples were calculated relative to this value and plotted using GraphPad Prism. For experiments involving cytochalasin D, the infected T24 bladder cells were treated with 0, 1, 5, or 10 μM cytochalasin D for 1 hour before adding NDs. The rest of the procedure was performed as described above.

### Statistical analyses

Data were analyzed using one-way or two-way analysis of variance (ANOVA) followed by the appropriate post-hoc tests with GraphPad Prism v7. *P*-value < 0.05 was considered to be statistically significant.

## Results

### Purification and characterization of 6 and 25 nm NDs

**Fig 1:**
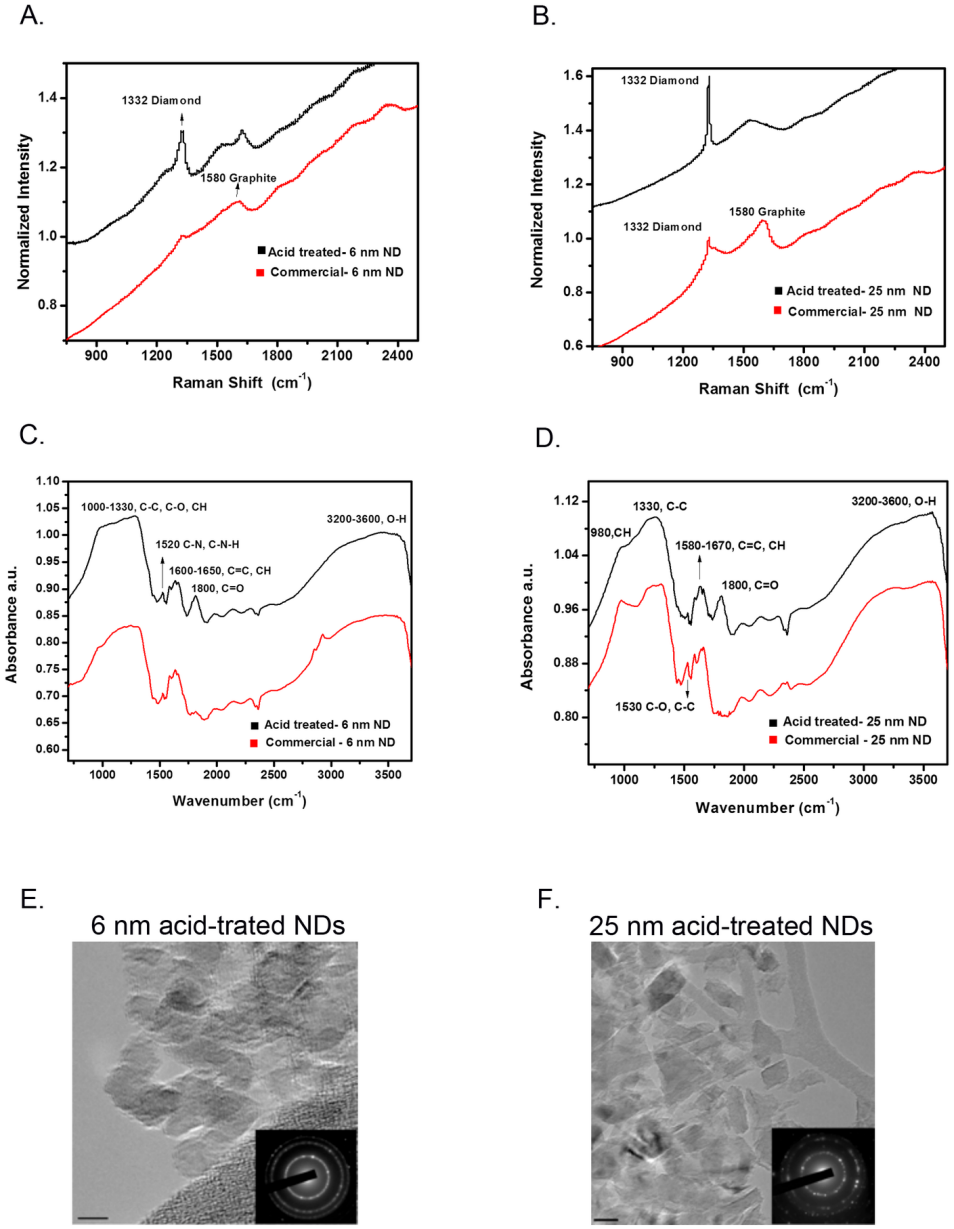
Purification and characterization of 6 nm and 25 nm nanodiamonds. Raman spectra of 6 nm (A) and 25 nm (B) commercial (black line) and acid-treated NDs (red line). FTIR spectra of 6 nm (C) and 25 nm (D) commercial (black line) and acid-treated NDs (red line). (E) Transmission electron micrographs from 6 nm and 25 nm acid-treated NDs. The diffraction patterns of the NDs are depicted in the inset of each image. Scale bar for 6 nm acid-treated NDs = 5 nm and scale bar for 25 nm acid-treated NDs = 20 nm.

The purity of commercially obtained 6 and 25 nm NDs was evaluated by Raman spectroscopy and both sizes of NDs showed the presence of graphite that is observed at 1580 cm^-1^ (Fig 1A). Following acid treatment, both sizes of NDs showed a strong diamond peak at 1332 cm^-1^ and a reduction of the graphite peak at 1580 cm^-1^ (Fig 1B) indicating the successful removal of most of the graphite and other carbon impurities around the diamond core. As described previously, this procedure resulted in a diamond yield of 97.51% and 98.25% for acid-treated 6 nm and 25 nm NDs, respectively [32]. On comparing the FTIR spectrum of acid-treated 6 and 25 nm NDs with the commercially obtained NDs, there was a strong carboxyl group band (C = O) at 1800 cm^-1^ that was observed only in the acid-treated NDs (Fig 1C and 1D). The acid-treated 6 nm NDs also showed a C-N and C-N-H related peak at 1520 cm^-1^. Zeta potential measurements of the NDs revealed that the surface of 6 nm acid-treated NDs was positively charged while 25 nm acid-treated NDs were negatively charged (Table 1). 6 nm NDs were more acidic than 25 nm NDs as observed by pH measurements, however, there were no drastic differences in the pH of the ND suspensions (Table 1). By TEM analysis, 6 nm NDs appeared more spherical in shape while 25 nm NDs appeared to be irregular in shape (Fig 1E). In summary, the differences in surface charges and functional groups may have an impact on the interactions of acid-treated 6 nm and 25 nm NDs with various biological systems.

**Table 1:** pH and zeta potential measurements of 6 nm and 25 nm commercial and acid-treated NDs.

NDs	pH	Zeta potential (mV)
Commercial 6 nm	5.21	+42 ± 6.47
Acid-treated 6 nm	5.71	+44 ± 6.21
Commercial 25 nm	6.16	-40 ± 4.32
Acid-treated 25 nm	6.54	-60 ± 5.21

### 6 nm NDs are less toxic to T24 bladder cells than 25 nm NDs

**Fig 2:**
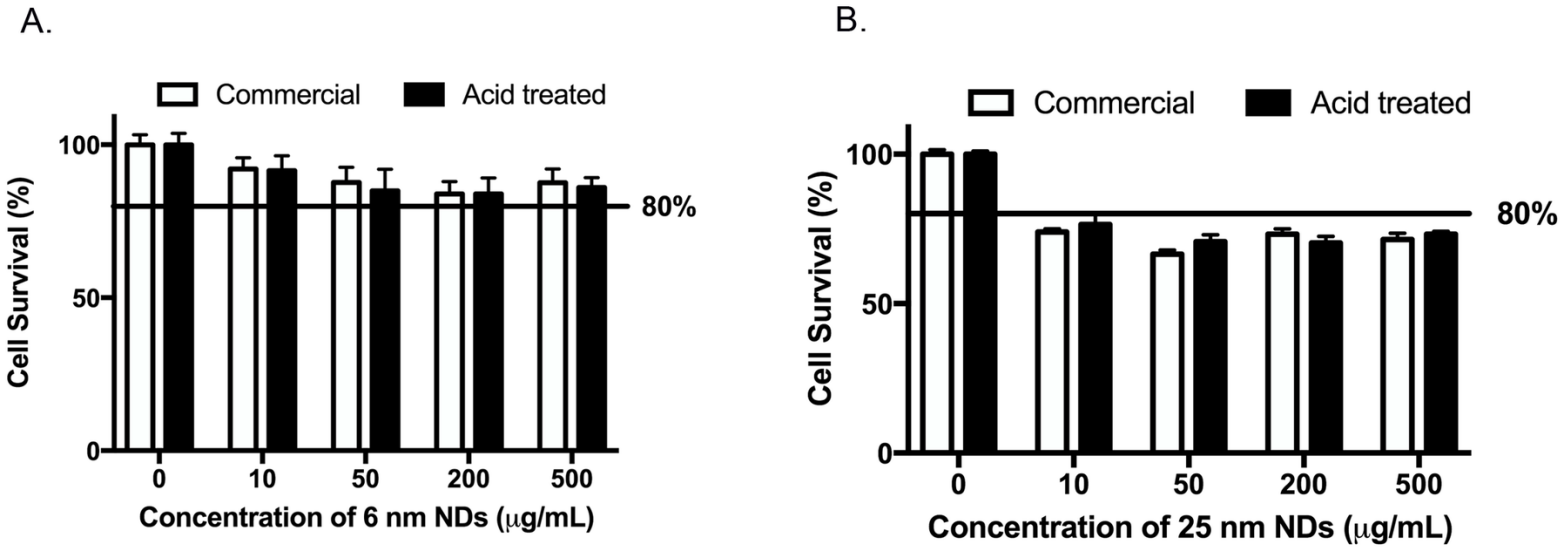
Cytotoxicity of 6 nm and 25 nm NDs in T24 cells. T24 cells were treated with 6 nm (A) or 25 nm (B) commercial (white bars) or acid-treated (black bars) NDs for 24 hours. Cytotoxic effects of NDs were evaluated by the MTT assay. Data are representative of at least three independent experiments and depicted as mean ± SEM. There were statistically significant (*P* < 0.05) decreases in the percentages of cell survival following treatment with NDs as compared to the corresponding 0 μg/mL sample as determined by 2-way ANOVA followed by Tukey’s multiple comparison’s test.

Nanodiamonds are known for displaying low levels of toxicity towards a variety of mammalian cell lines [17]. The extent of cytotoxicity depends on the size as well as functional groups present on the surface of the NDs. In order to determine if acid treatment altered the cytotoxicity of the NDs, T24 bladder cells were treated with different concentrations of commercial and acid treated 6 nm and 25 nm NDs for 24 hours. To assess the extent of cytotoxicity, we measured the ability of the cells to metabolize tetrazolium salts to formazan. There was a significant decrease in cell viability upon treatment with NDs as compared to untreated sample (0 μg/mL). There was no significant difference in cell viability between the commercial or acid-treated samples (Fig 2). However, the cell viability was more than 50% even at the highest concentration (~85% for 6 nm and ~73% for 25 nm at 500 μg/mL). When we compared the cytotoxicity of acid-treated 6 nm and 25 nm NDs (Table 2), 6 nm acid-treated NDs showed significantly greater cell viability than 25 nm acid-treated NDs. Thus, 6 nm acid-treated NDs are less toxic to T24 bladder cells than 25 nm acid-treated NDs.

**Table 2:** Comparison of cytotoxicity of acid treated 6 nm and 25 nm NDs.

Concentration of NDs (μg/mL)	Cell survival (%)	
	6 nm acid	25 nm acid
0	100.0 ± 1.0	100.0 ± 0.8
10	91.6 ± 1.5	76.4 ± 3.7**
50	86.1 ± 1.6	70.7 ± 2.3**
200	82.1 ± 2.3	70.9 ± 2.1**
500	85.4 ± 1.0	73.2 ± 0.9**

**P > 0.0001 compared to corresponding concentration of 6 nm acid-treated NDs

### 6 nm NDs can kill extracellular and intracellular UPEC more effectively than 25 nm NDs

**Fig 3:**
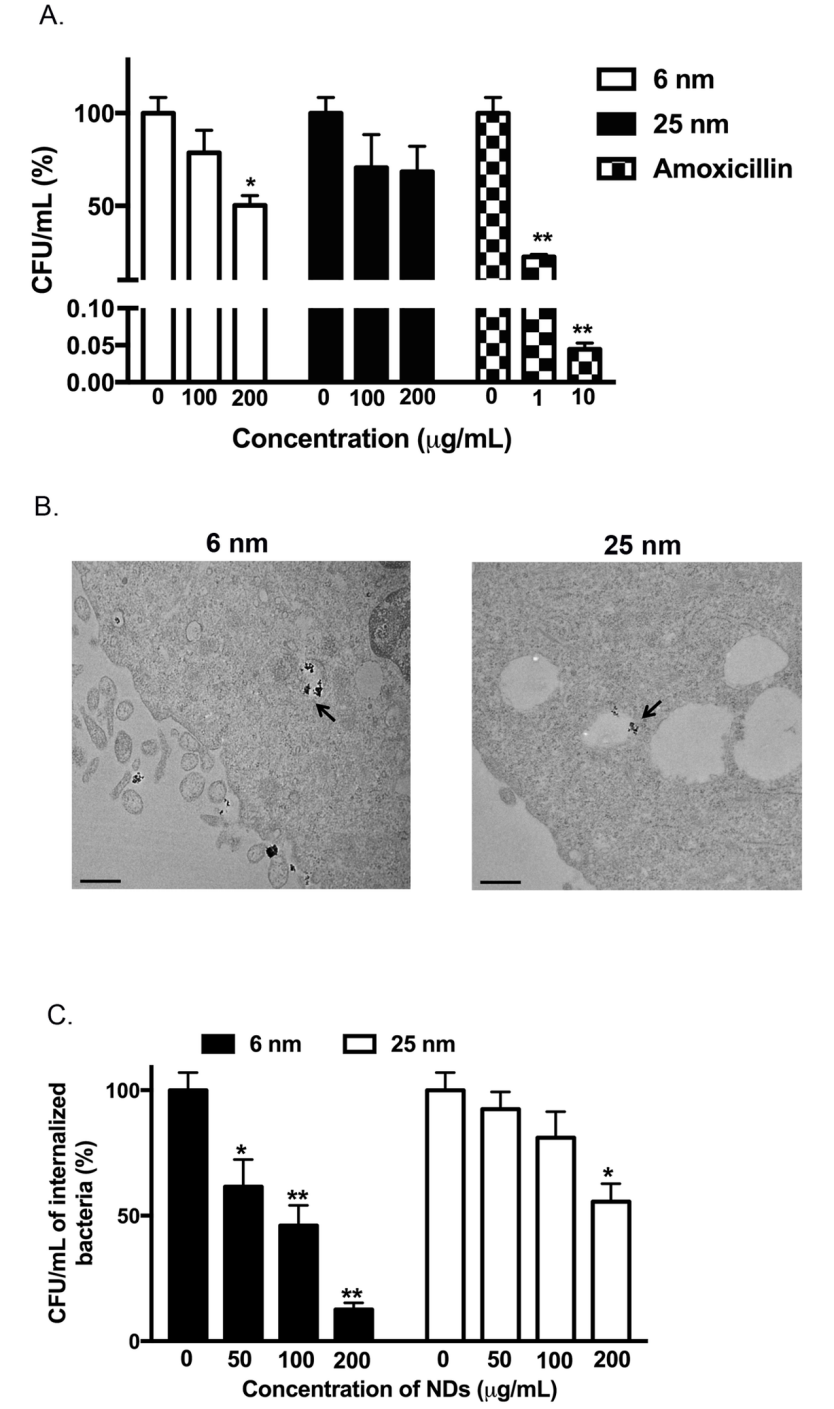
Acid-treated 6 nm NDs can kill extracellular and intracellular *E*. *coli*. (A) *E*. *coli* was treated with different concentrations of acid treated 6 nm or 25 nm NDs. Treatment with amoxicillin was used as a positive control. Following incubations, the samples were plated on sterile Luria agar and the colony forming units (CFU)/mL were enumerated. The CFU/mL for the 0 μg/mL sample was determined 100%. The CFU/mL counts for ND treated samples were determined relative to the 0 μg/mL sample. Data is represented as mean ± SEM. *P<0.*001*, **P<0.*0001* vs 0 μg/mL was determined by one-way ANOVA followed by Tukey’s multiple comparisons test. (B) T24 cells were treated with 6 nm or 25 nm acid-treated NDs for 4 hours and analyzed by TEM. NDs are indicated by arrows. Scale bar = 500 nm. (C) T24 cells were infected with *E*. *coli*, followed by treatment with gentamicin to kill extracellular bacteria. Following gentamicin treatment, the infected T24 cells were treated with 6 nm or 25 nm acid treated NDs for 24 hours. The cells were then lysed and intracellular *E*. *coli* were enumerated. The CFU/mL for the 0 μg/mL sample was determined 100%. The CFU/mL for ND treated samples were determined relative to the 0 μg/mL sample. Data are representative of at least three independent experiments and depicted as mean ± SEM. *P<0.*01*, **P<0.*0001* compared to 0 μg/mL of the corresponding acid-treated ND was determined by two-way ANOVA followed by Tukey’s multiple comparisons test.

We evaluated the antibacterial activity of the acid-treated 6 and 25 nm NDs by treating UPEC with the NDs for 2 hours. Acid-treated 6 nm NDs displayed significant antibacterial effects on UPEC as compared to acid-treated 25 nm NDs at a concentration of 200 μg/mL (Fig 3A). Amoxicillin was used as a positive control for antibacterial activity. Since UPEC internalize into host cells using the endocytic machinery, we determined the ability of acid-treated NDs to associate and internalize into T24 bladder cells by TEM. T24 bladder cells were treated with NDs for 4 hours, followed by fixation and processing for TEM imaging. Acid-treated 6 and 25 nm NDs were found not only to associate with the T24 bladder cells, but they were also internalized into the cells in endocytic vesicles (Fig 3B). Since we could observe NDs in the endosomes of cells, we determined if NDs could kill intracellular bacteria that also traffick in endosomes. T24 bladder cells were infected with UPEC that are capable of invading into bladder cells to form niches. The extracellular bacteria were killed by gentamicin that is not cell permeable and hence is ineffective against intracellular bacteria. The infected bladder cells were treated with different concentrations of acid-treated 6 and 25 nm NDs for 24 hours. Upon enumerating the intracellular bacteria after ND treatment, we found that there was a significant dose dependent decrease in intracellular bacteria when the T24 bladder cells were treated with acid-treated 6 nm NDs (Fig 3C). When infected cells were treated with acid-treated 25 nm NDs, there was a significant reduction in intracellular UPEC only at the highest concentration (200 μg/mL). Furthermore, acid-treated 6 nm NDs were significantly better at killing intracellular UPEC than 25 nm NDs at all concentrations that were tested (Table 3). Since 6 nm NDs showed lower cytotoxicity and better ability to kill extracellular and intracellular UPEC, we utilized 6 nm NDs for further studies.

**Table 3:** Comparison of % of internalized bacteria in cells treated with 6 nm or 25 nm NDs.

Concentration (μg/mL)	CFU/mL of internalized bacteria (%)	
	6 nm (mean ± SEM)	25 nm (mean ± SEM)
0	100 ± 7.0	100 ± 7.0
50	62 ± 10.8	93 ± 6.9*
100	46 ± 8.0	81 ± 10.3**
200	13 ± 2.5	56 ±7.2**

*P > 0.05 compared to corresponding concentration of 6 nm acid-treated NDs;

**P > 0.01 compared to corresponding concentration of 6 nm acid-treated NDs was determined by two-way ANOVA followed by Sidak’s multiple comparisons test.

### 6 nm NDs are internalized in T24 bladder cells by 2 hours

**Fig 4:**
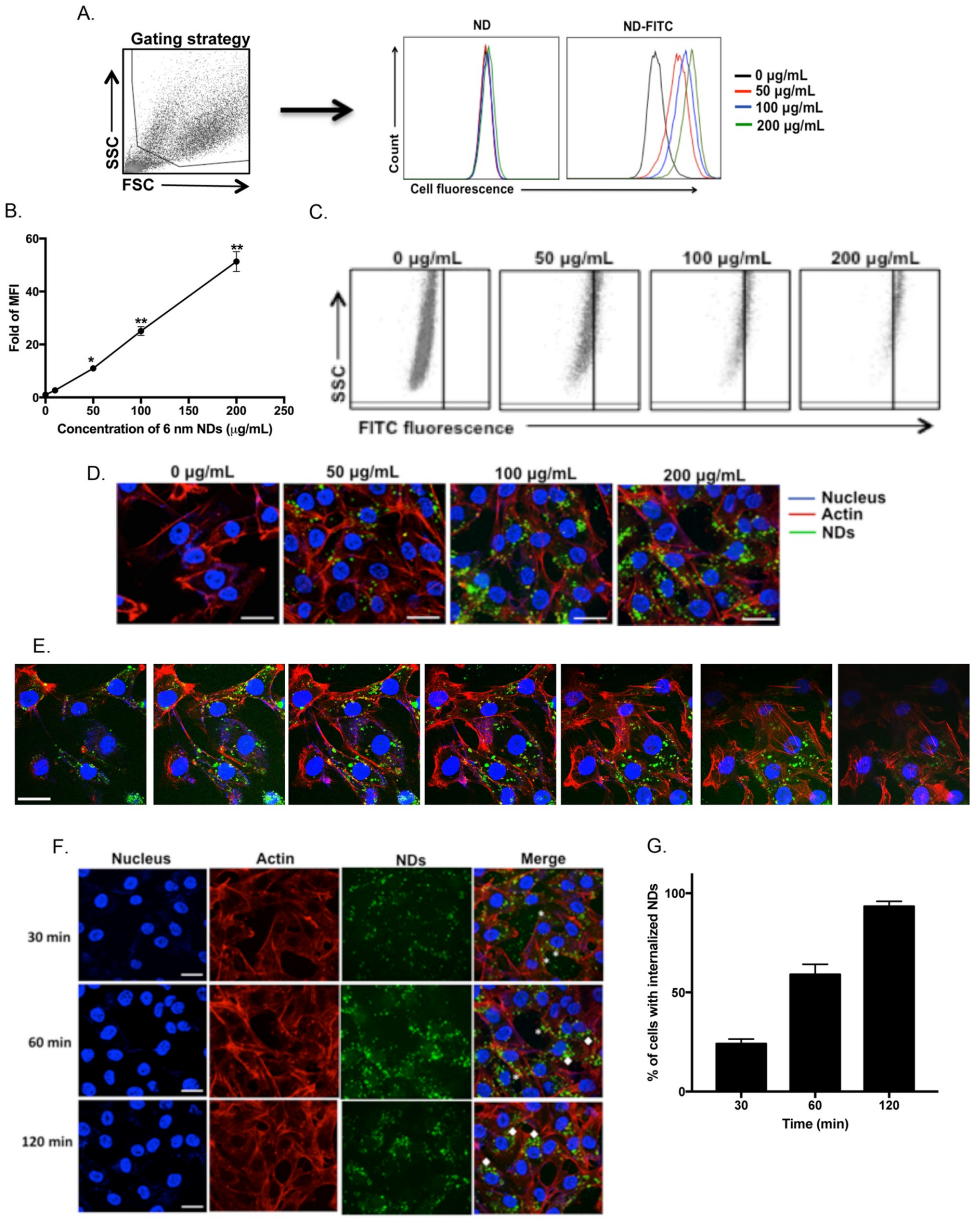
Acid-treated 6 nm NDs are internalized in T24 cell by 2 hours. T24 cells were treated different concentrations of 6 nm acid-treated FITC labeled of unlabeled NDs for 1 hour. The cells were washed and analyzed by flow cytometry. The gated cells were evaluated for FITC fluorescence and the overlayed histograms (A) are depicted. The experiment was performed at least three independent times in duplicates. The result of one representative experiment is depicted. (B) The mean fluorescence intensity (MFI) for samples treated with FITC-labeled NDs was evaluated. The MFI of the untreated sample (μg/mL) was considered 1 and the MFI for all other samples were normalized to the untreated sample and plotted as mean ± SEM. Data are representative of at least three independent experiments. *P<0.*01*, **P<0.*0001* was determined by one-way ANOVA followed by Tukey’s multiple comparisons test. (C) Scatter plots of Side scatter (SSC) vs FITC fluorescence of gated cells are depicted. The experiment was performed at least three independent times in duplicates. The result of one representative experiment is depicted. (D-G) T24 bladder cells were treated with different concentrations of FITC labeled 6 nm NDs for 1 hour (D-E) or with 100 μg/mL for different periods of time (F). The cells were fixed and permeabilized. The nuclei of the cells were stained with DAPI and actin filaments were stained with phalloidin conjugated to Alexa fluor 660. The cells were then observed by confocal microscopy. The experiment was performed at least three independent times and the result of one representative experiment is depicted. To determine the internalization of NDs in T24 bladder cells (E), z-stack analysis was performed and a few representative stacks of one image are shown. For the kinetic analysis (F), T24 bladder cells were treated with 100 μg/mLof FITC-labeled 6nm NDs for different periods of time. Z-stack analysis was performed and one representative image from the middle of the stack is depicted below. NDs associated with the cell membrane (*) or internalized in the cells (◆) are shown. Scale bar = 25 μm. The % of cells with NDs internalized was plotted (G) as mean ± SEM.

In order to better characterize the interactions of acid treated 6 nm NDs with T24 bladder cells, we labeled the NDs with fluorescein isothiocyanate to enable efficient detection of the NDs by flow cytometry and confocal microscopy. T24 bladder cells were treated with different concentrations of unlabeled acid-treated or FITC-labeled acid-treated 6 nm NDs for 1 hour at 37°C. The associations between NDs and T24 bladder cells were quantified by flow cytometry by gating the T24 bladder cells based on their forward and side scatter properties (Fig 4A). There was a dose dependent increase in the mean fluorescence intensity (FITC fluorescence) of the gated T24 bladder cells that were treated with FITC-labeled NDs (Fig 4A and 4B). Similarly, there was a dose dependent increase in the number of cells interacting with NDs (Fig 4C). We confirmed these findings by confocal microscopy. Upon staining the nucleus and actin filaments of T24 bladder cells, we could observe NDs interacting with T24 cells in a dose-dependent manner (Fig 4D) and by performing z-stack analysis, the NDs were found to be internalized in T24 bladder cells (Fig 4E). Since TEM analysis revealed that the NDs were present in endocytic vesicles, we performed a kinetic analysis to determine when the NDs were internalized into T24 bladder cells. T24 bladder cells were treated with NDs for different periods of time and our findings indicate that NDs interact with the plasma membrane of T24 bladder cells in 30 minutes and by 2 hours are efficiently internalized (Fig 4F and 4G).

### Internalization of 6 nm NDs are required for effective killing of intracellular bacteria

**Fig 5:**
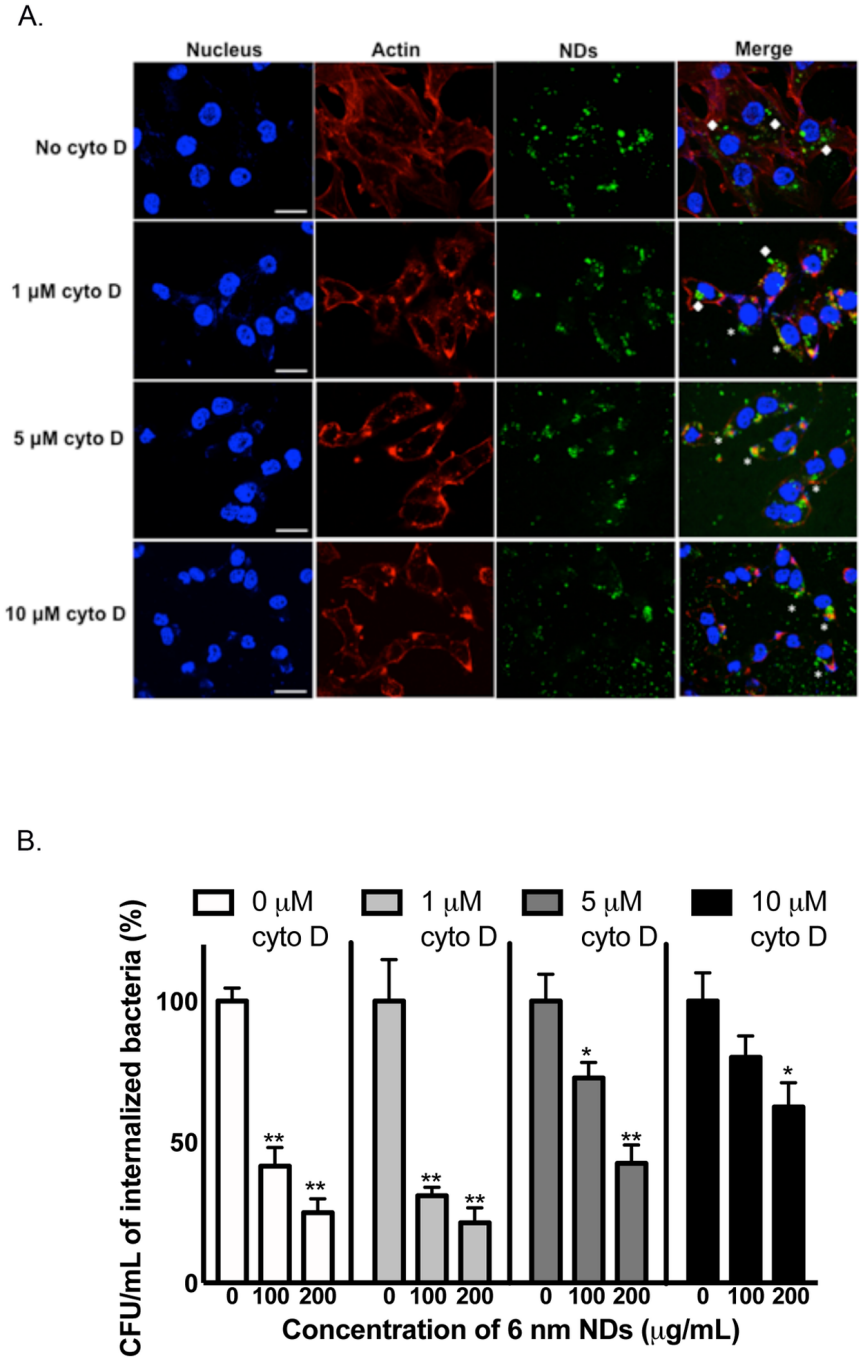
Internalization of acid-treated 6 nm NDs are required for efficient killing of intracellular bacteria. (A) T24 cells were treated with different concentrations of cytochalasin D for 1 hour followed by treatment with 100 μg/mL of NDs for 2 hours. The cells were fixed, permeabilized and the nuclei of the cells were stained with DAPI. Actin was stained with phalloidin conjugated to Alexa fluor 660. The cells were then observed by confocal microscopy and subjected to z-stack analysis to determine internalization of NDs. NDs associated with the cell membrane (*) or internalized in the cells (◆) are shown. The experiment was performed at least three independent times and the result of one representative experiment is depicted. Scale bar = 25 μm. (B) T24 cells were infected with *E*. *coli*, followed by treatment with gentamicin to kill extracellular bacteria. After gentamicin treatment, the infected T24 cells were treated with different concentrations of cytochalasin D for 1 hour followed by treatment with 6 nm or 25 nm acid treated NDs for 24 hours. The cells were then lysed and intracellular *E*. *coli* were enumerated. The cfu counts for the untreated sample was determined 100%. The CFU counts for ND treated samples were normalized to the 0 μg/mL sample. Data are representative of at least three independent experiments and depicted as mean ± SEM. *P<0.*05*, **P<0.*0001* compared to 0 μg/mL of the corresponding acid-treated ND sample was determined by two-way ANOVA followed by Tukey’s multiple comparisons test.

We determined if NDs utilize actin filaments to enter T24 bladder cells by treating cells with different concentrations of cytochalasin D (an inhibitor of actin polymerization) for 1 hour before adding the NDs. We found that treatment with 1 μM of cytochalasin D did not affect the internalization of NDs greatly but treatment with 10 μM of cytochalasin D resulted in majority of NDs localizing on the cell surface. Thus 10 μM of cytochalasin D was most effective in preventing the internalization of NDs into T24 bladder cells (Fig 5A) thereby confirming the actin-dependent uptake of NDs. We further assessed if internalization of NDs was critical for killing of intracellular UPEC by treating infected T24 bladder cells with different concentrations of cytochalasin D for 1 hour prior to treatment with nanodiamonds. We observed a dose dependent reduction in the ability of NDs to kill intracellular UPEC in the presence of cytochalasin D (Fig 5B). ND treatment in the presence of 1 μM cytochalasin D could kill intracellular UPEC efficiently. However, ND treatment in the presence of 10 μM cytochalasin D could not effectively reduce the number of intracellular UPEC. These findings suggest that internalization of NDs is a crucial step toward the killing of intracellular UPEC.

## Discussion

Urinary tract infections (UTIs) are one of the most prevalent bacterial infections that affect children as well as adults [9, 37]. Uropathogens, including UPEC, have developed mechanisms to evade the actions of antibiotics through the acquisition of antibiotic resistance genes or by hiding in host tissues that enable them to internalize and persist in the urinary tract to cause recurrent UTI infections [38]. UPEC can invade into the superficial epithelial umbrella cells of the bladder by binding to different cell surface receptors [39–41] and once they are internalized in the umbrella cells, they multiply and form IBCs [42]. The bacteria can then disperse from IBCs and infect neighboring host cells leading to recurrent UTIs. UPEC can also invade into the underlying transitional epithelial cells and form QIRs [43]. When the transitional epithelial cells differentiate to umbrella cells, the bacteria become active and cause recurrent UTIs. Thus, by forming IBCs and QIRs, UPEC are able to evade immune surveillance and the action of antibiotics. Furthermore, the prevention of UTIs have been hampered by our poor understanding of the immune mechanisms functioning in the urinary tract as well as the absence of an effective vaccine [44]. Recently, a novel vaccine against extraintestinal *E*. *coli* has been tested for the prevention of UTIs but until the safety and efficacy of the vaccine is confirmed, UTIs will remain a public health concern [45]. Hence, it is imperative to design therapeutics that have the ability to penetrate into the host bladder epithelial cells to disrupt these IBCs and QIRs.

Various nanomaterials are being tested for antibacterial properties against Gram-positive and Gram-negative bacteria for the purpose of treating infections. Recently, carbon-based nanoparticles that include fullerenes, nanotubes and nanodiamonds among others, have also been tested for antibacterial applications. There are contradictory reports on the antibacterial effects of fullerenes [46, 47], but carbon nanotubes have been reported to display antibacterial properties [48]. Kang et al., reported that single-walled carbon nanotubes showed greater antibacterial activity than multi-walled carbon nanotubes by inducing the expression of stress-related genes in *E*. *coli* [49]. In the current study we found that 6 nm acid-treated NDs displayed significant antibacterial properties at a concentration of 200 μg/mL against a clinical strain of UPEC. These findings are in contrast with a study performed by Beranova et al., who showed that air-oxidized 5 nm detonation NDs were antibacterial only at concentrations higher than 500 μg/mL in the *E*. *coli* K12 strain [23] and Chatterjee et al., who reported that 5 nm detonation NDs that were purified by chemical treatment showed modest antibacterial activity towards *E*. *coli* HB101 strain at a concentration of 100 μg/mL [25]. The discrepancies observed could be due to the difference in the strains of *E*. *coli* that were used. We also propose that differences in size and surface functional groups influence the activity of NDs with the bacteria. An elegant study by Wehling et al., using 5 nm detonation NDs with different surface functional groups, showed that only certain types of NDs were antibacterial. They further demonstrated that NDs that possessed an anhydride group on their surface displayed significant antibacterial activity against *E*. *coli* K12 strain while all other NDs showed minimal antibacterial activity [24]. The 6 and 25 nm NDs that we employed in the current study were synthesized by detonation and the HPHT method, respectively [30]. Our characterization studies revealed that both types of NDs were effectively purified by a previously described acid-treatment method [32]. Both acid-treated NDs showed the presence of alcoholic and carboxylic groups on their surface and the 6 nm NDs showed the additional presence of C-N bonds. The acid-treated NDs also had different surface charges with the 6 nm NDs being positively charged while the 25 nm NDs being negatively charged. We speculate that these physico-chemical differences are responsible for the altered antibacterial activity observed between the acid-treated 6 vs 25 nm NDs.

In addition to differences in antibacterial activity, the NDs also showed differences in mediating cytotoxicity in T24 bladder cells. Our findings indicate that acid-treated 6 and 25 nm NDs, enabled more than 70% of T24 bladder cells to survive at a concentration of 500 μg/mL. Additionally, the 6 nm NDs were significantly less cytotoxic (>80% viability) to T24 bladder cells as compared to 25 nm NDs (>70% viability). NDs have been shown to display less cytotoxicity than other nanoparticles like nanotubes and graphite. Schrand et al., showed that NDs were more biocompatible than single- or multi-walled nanotubes and carbon black in a neuroblastoma cell line and rat alveolar macrophages [18]. NDs were also less toxic than nanographite making them more advantageous for human-based therapeutic applications [17]. Similar to antibacterial activity, the differences in levels of cytotoxicity between the 6 and 25 nm acid-treated NDs can be attributed to the differences in size and surface functional groups. Our results on 6 nm NDs showed least cytotoxicity on bladder cells. Some of the previous studies on NDs have shown that size is the main determinant of nanoparticle cytotoxicity as it determines the bio-distribution of nanoparticles delivery system. In fact, smaller size ultra-crystalline diamond films used in *in vitro* and *in vivo* models were found to reduce host inflammatory response by down-regulating cytokine and chemokine responses [50, 51].

TEM imaging revealed that acid-treated NDs were present in endocytic vesicles of T24 bladder cells. During UTIs, UPEC also invade into bladder cells through the endocytic pathway and hence traffick through endosomes [52]. We hypothesized that NDs will be internalized in human bladder cells and facilitate the killing of intracellular UPEC. We observed that 6 nm acid-treated NDs were significantly better in reducing the number of intracellular UPEC than 25 nm NDs in T24 bladder cells thereby validating our hypothesis. This is the first study that demonstrates the ability of plain NDs to internalize into human bladder epithelial cells and kill intracellular UPEC. There has been a report stating that NDs conjugated with glycans like tri-thiomannoside clusters prevents bacterial biofilm formation and inhibits adhesion of *E*. *coli* to T24 bladder cells, but the ability of NDs to kill intracellular *E*. *coli* was not determined in that study [53]. Among other nanoparticles, there is a report demonstrating that mesoporous silica nanoparticles loaded with rifampin could kill intracellular *M*. *tuberculosis* present in macrophages with greater efficacy than free rifampin. However the study also showed that plain silica nanoparticles were ineffective [54]. Thus, our finding that plain NDs could decrease the bacterial load in infected mammalian host cells is unique and novel.

It is now well established that nanostructures of different sizes and shapes interact differently with the cells at molecular level [55]. The binding and activation of membrane receptors and subsequent protein expression has been shown strongly depend on nanoparticle size. To validate our studies of NDs associating with T24 bladder cells, we characterized the interaction of 6 nm acid-treated NDs with T24 bladder cells by flow cytometry and confocal microscopy. The NDs bound to T24 bladder cells in a dose dependent manner and we could observe the NDs inside the T24 bladder cells 2 hours post-treatment by confocal microscopy. There are studies demonstrating the internalization of NDs into other mammalian cell lines like SH-SY5Y, HeLa, NIH/3T3 etc. [16, 56, 57]. NDs have been shown to internalize into mammalian cells predominantly by clathrin-dependent endocytosis but can also be taken up by macropinocytosis [26, 58]. In the current study, the internalization of 6 nm acid-treated NDs in T24 bladder cells was dependent on actin polymerization. Furthermore, the internalization of 6 nm NDs in T24 bladder cells was critical for the ability of the NDs to reduce intracellular UPEC in infected T24 bladder cells. Thus, interactions of NDs with the cell surface was not enough to reduce the intracellular bacterial load. The identity of extracellular receptors that are involved in the internalization of NDs are not known but there have been reports indicating that NDs can bind to proteins in serum that facilitate their uptake by mammalian cells [59].

Based on our findings in bladder cells, 6 nm acid-treated NDs bind, internalize and are able to kill intracellular UPEC and thus may serve as future platforms for newer antimicrobial therapeutic agents. Internalized 6nm NDs may induce signaling and gene changes such as oxidative stress, cell cycle regulation and inflammation to inhibit *E*. *coli* survival in the endosomes or bacteria escaping to the cytosol.

Future aim of our studies are to test targeted delivery of these 6 nm NDs using intravenous route using thermosensitive liposome technology [60] or deliver NDs intravesically in to the bladder by urethral catheterization to treat resistant UTI in an *in vivo* mouse model. We will assess the antimicrobial effects of the NDs in the infected bladder and also effects in other major organs such as liver, spleen, and kidneys. Many anti-cancer drugs for bladder cancer are delivered intravesically and thus, it is a well-established route for drug delivery in addition to intravenous delivery. Further, NDs are being developed for bio-imaging and drug delivery. These studies have reported that small size NDs injected via the intravenous route go to liver, spleen and kidneys but cause minimal toxicity and are finally excreted in the urine [61].

We are performing studies for loading NDs with appropriate antimicrobial agents so that we can generate more efficient therapeutic agents for the dual purpose of targeting intracellular uropathogens and delivering low doses of antimicrobial agents. We are also investigating the therapeutic efficacy of NDs in treating UTIs in a preclinical *in vivo* model of UTI [33]. It has been shown that intravenous administration of NDs resulted in a low *in vivo* toxicity and excretion of the NDs in the urinary bladder [62, 63]. Hence, we are confident that administration of 6 nm acid-treated NDs can serve as novel therapeutics for treating UTIs.
